# Soft X-ray Microscopy in Cell Biology: Current Status, Contributions and Prospects

**DOI:** 10.32607/actanaturae.26551

**Published:** 2023

**Authors:** S. A. Golyshev, E. P. Kazakov, I. I. Kireev, D. G. Reunov, I. V. Malyshev

**Affiliations:** Belozersky Institute of Physico-Chemical Biology, Lomonosov Moscow State University, Moscow, 119992 Russian Federation; Institute of Physics of Microstructures RAS, Nizhny Novgorod, 603950 Russian Federation

**Keywords:** X-ray microscopy, cell biology, soft X-ray, water window, cryotomography

## Abstract

The recent advances achieved in microscopy technology have led to a significant breakthrough in biological research. Super-resolution fluorescent microscopy now allows us to visualize subcellular structures down to the pin-pointing of the single molecules in them, while modern electron microscopy has opened new possibilities in the study of protein complexes in their native, intracellular environment at near-atomic resolution. Nonetheless, both fluorescent and electron microscopy have remained beset by their principal shortcomings: the reliance on labeling procedures and severe sample volume limitations, respectively. Soft X-ray microscopy is a candidate method that can compensate for the shortcomings of both technologies by making possible observation of the entirety of the cellular interior without chemical fixation and labeling with an isotropic resolution of 40–70 nm. This will thus bridge the resolution gap between light and electron microscopy (although this gap is being narrowed, it still exists) and resolve the issue of compatibility with the former, and possibly in the near future, the latter methods. This review aims to assess the current state of soft X-ray microscopy and its impact on our understanding of the subcellular organization. It also attempts to look into the future of X-ray microscopy, particularly as relates to its seamless integration into the cell biology toolkit.

## PRINCIPLES OF SXM AND HOW IT COMPARES WITH OTHER TYPES OF MICROSCOPY

The modern technologies used in microscopic research in biology make it possible to address a wide range of problems: from monitoring of the development of the whole embryos, through the localization of single molecules in a cell, to direct visualization of the structure of macromolecules in their native state [1, 2, 3]. The development of both light and electron microscopy is constantly expanding the range of possibilities for researchers; however, despite all the successes achieved so far, both approaches retain their fundamental limitations.

**Fig. 1:**
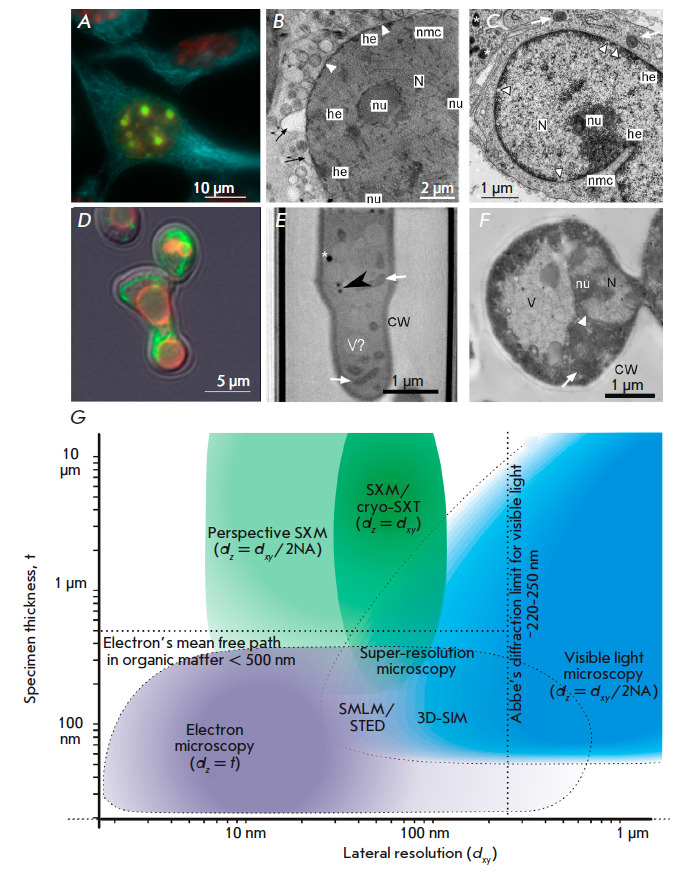
Soft X-ray microscopy in comparison with visible light and electron microscopy. (*A*–*C*) – mammalian cells (mouse fibroblasts). (*A*) – fluorescent micrograph of whole chemically fixed mouse cells *in vitro*: blue – microtubules, fluorescent anti-α-tubulin antibodies; green – newly replicated DNA, click-reaction with ethynyl-deoxyuridine; red – nuclei, DNA-binding fluorescent dye DAPI. (*B*) – tomographic slice of cryofixied cell by cryo-SXT: N – nucleus, nu – nucleoli; he – heterochromatin, nmc – nuclear membrane channel, arrowheads – nuclear envelope, black arrows – outer nuclear membrane blebs (adapted with modifications from [11] CC 4.0 BY). (*C*) – EM micrograph of aldehyde-fixed heavy metal- stained cell: mouse connective tissue, ultrathin section: white arrow – mitochondria; * – lipid droplets. (*D*–*F*) – yeasts* S. cerevisiae*. (*D*) – fluorescent micrograph of a whole cell (zygote): green – mitochondria, chimeric mitochondrial protein Idh1-GFP; red – vacuolar membrane, FM4-64 fluorescent dye; cell outline – differential interference contrast (photo kindly provided by Knorre D.A.). (*E*) – tomographic slice of cryofixied cell by cryo-SXT: V – vacuole, CW – cell wall; black arrowhead – structure used for correlation of images, possibly – small lipid droplet, (adapted with modifications from [15] CC0 1.0). (*F*) – EM micrograph of aldehyde-fixed heavy metal-stained cell, ultrathin section. (*G*) – Soft X-ray, visible light and electron microscopy positions in a “resolution space.” Color intensity represents the scale in which each method is mostly demanded in the studies of the cellular structure and functions. NA – numeric aperture, the light-gathering capacity of the microscopes’ lens which, in combination with the wavelength (λ), is the determinant of the critical resolution: dxy = 0,61 × λ/NA [4]. Photos A, C, F were taken by the authors of the review

Light microscopy is developing towards improving fluorescent methods, whose main advantage is the high contrast of the resulting image, in combination with the highest selectivity and sensitivity of fluorescent labeling methods [4]. The exploitation of these features has made it possible to bypass the diffraction limit for the resolving power of light microscopes. In the widely used methods of super-resolution light microscopy, a resolution of about 30 nm is routinely achieved [1]. However, the advantages of fluorescence microscopy are also its limitations. While allowing the observation of labeled molecules and the structures formed by them, a fluorescent microscope does not show components that do not carry labels (*Fig. 1A,D*). It is difficult to use more than three or four fluorescent labels in one experiment, and “optical” contrast methods (phase-contrast and differential interference contrast microscopy) are significantly inferior to fluorescence in terms of resolving power (*Fig. 1A,D*). Resolution anisotropy is an important limitation of most fluorescent methods aimed at maximizing the resolving power: in the axial direction, the resolving power is approximately two times worse than that in the focal plane.

A fundamental limitation of transmission electron microscopy is the mean free path of an electron in a substance, which at maximum does not exceed 300–500 nm; therefore, ultrathin sections of the object under study, with a thickness comparable to the mean free path of electrons, need to be prepared for TEM studies [5]. Chemical fixation is necessary for meeting this requirement, which alters the structure and composition of the sample [5, 6]. The need for examining a series of sections, sometimes up to hundreds of slices, makes it difficult to study the three-dimensional organization of a cell whose dimensions are two orders of magnitude greater than the allowable thickness of the sections. Various approaches are used to overcome this limitation, each being a compromise between the volume under study, resolving power, and labor costs [7].

There are even more limitations for cryo-electron tomography methods, whose main advantage is that the native structure and composition of the cell remain preserved. The peculiarities of sample preparation force researchers to focus on the naturally thin cell portions or prepare a single slice (a lamella 100–500 nm thick) from the cell using ion beam milling. Therefore, the main area of application of cryo- EM tomography is in the analysis of macromolecular complexes in their native environment [8].

The role of a method that to some extent allows one to overcome the aforementioned limitations is claimed by cryo soft X-ray tomography (cryo-SXT), which currently is the most advanced branch of biological soft X-ray microscopy (SXM) [9]. The characteristics of this method are as follows: (1) it provides resolution of ~ 50 nm, and (2) it can be used to examine objects approximately 10 μm in thickness without the need to prepare sections (3) in the near-native vitrified state and (4) without using additional contrast or selective labeling to identify a given set of subcellular structures.

The objectives of this review were to assess the principles of the method, the instrumental basis and contribution of soft X-ray microscopy to cell biology, identify the research areas for which the features of SXM are most suitable, and to discuss the prospects for further development and implementation of SXM in cell biology.

SXM uses X-ray radiation with a photon energy of ~ 500 eV (wavelengths from 2.3 to 4.4 nm), corresponding to the so-called “water transparency window.” In this energy range, oxygen atoms, and therefore water molecules, weakly absorb X-ray photons (depending on the wavelength, absorption ranges from 10 to 40% in a 1-μm thick layer of water). Therefore, a sample of up to 15 µm in thickness is suitable for research [10, 11, 12]. Under these conditions, atoms of carbon and other light elements become efficient generators of absorption contrast. Most modern synchrotrons can generate X-rays in this energy range with high luminous flux intensity [13].

An SXM station consists of a synchrotron source of X-ray photons, a monochromator, a focusing system that illuminates the output aperture (microscope light source), and the microscope *per se*, where either a zone plate or the so-called “capillary condenser” is used as a condenser. The condenser projects a reduced image of the synchrotron output aperture onto the sample. The sample is placed on a cryogenically cooled high-tilt stage with a precision positioning mechanism. A zone plate is used as an objective lens, projecting an enlarged image of the sample on the digital detector. The optical paths of such stations make it possible to generate images with a depth of focus of 1.5–10 µm, and the resolution of such installations can range from 30 to 70 nm [10, 13, 14]. Interchangeable zone plate lenses allow one to choose between depth of focus and resolving power [15].

With such a combination of the optical path parameters and thickness of the object, the image will therefore inevitably represent the sum of the projections of all intracellular structures, which is almost inaccessible for visual deciphering. Angular tomography becomes the most productive way to extract morphological data from such an image [16]. The high brightness of the synchrotron radiation source makes it possible to extract the necessary amount of data for tomographic reconstruction in a reasonable amount of time

Meanwhile, soft X-ray photons do not propagate in air, which requires placing all optical elements of the installation and the specimen in vacuum [17]. Overcoming this technical difficulty was facilitated by advances in the instrumental base of cryo-electron microscopy: the advent of automated devices for cryofixation of biological objects via ultra-fast freezing by plunging them into liquid ethane. This way, water does not crystallize but becomes an amorphous solid: vitrifies (“vitrum” from Latin “glass”) [18]. Such a sample, when its temperature is maintained at –180–100°C, is stable under vacuum and resists irradiation due to constant heat removal.

The second contribution of EM to the development of cryo-SXT was the creation of goniometric or tilting cryo-cooled sample holders for equipping cryo-EMs. These devices were adapted for use at some SXM stations intended for biological research, which consolidated the sample preparation procedure up to the use of standardized “grids” for EM in SXM instruments, but also introduced limitations inherent in EM tomography: the progressive increase in the effective sample thickness with an increasing tilt angle and the fact that it becomes shaded by holder structures at high tilts [10–13]. A unique feature of the SXM station at the ALS synchrotron (USA) is a fully rotating capillary sample holder, manufactured specifically for this installation and allowing isotropic resolution to be achieved without the distortions caused by tilt angle limitations [10, 19]. Vitrification of a sample by immersion using an automated device is a quick process, although it requires a certain level of skill, and preparing a sample for cryo-SXT examination takes about four hours, even if the design of a sample holder device is particularly complex [19].

The image of a cell obtained using cryo-SXT is very similar to a low-magnification electron micrograph [9, 16, 20] (*Fig. 1*); therefore, software packages for working with EM are suitable for reconstructing the volume from tilt series, for segmentation, and for subsequent data analysis [12, 13, 20]. Specialized tools are also being developed for working with cryo-SXT data, performing image restoration, and increasing their information yield [21], thus lightening the most operator-dependent steps: segmentation of three-dimensional data, as well as isolation of the contours and surfaces of organoids from the array of “voxels” [22, 23].

Scanning transmission X-ray microscopes (STXM), operating in the soft X-ray range, are less commonly used by biologists. In these devices, the sample is placed on a scanning stage, which displaces it with respect to a finely focused beam, and the image is generated based on changes in the brightness of the passing beam from point to point, measured by a single- pixel detector [24, 25].

Hence, SXM, especially in the implementation of cryo-SXT, is a mature method (in terms of the development of its technical base) focused on solving biological tasks, operating on the cellular scale, and holding a special place at the intersection of the capabilities of light and electron microscopy (*Fig. 1*).

## APPLICATIONS OF SX MICROSCOPY IN CELL BIOLOGY

Müller et al. [9] presented a catalog of images of intracellular structures recorded using cryo-SXT, such as the nuclear envelope, the nucleolus, mitochondria, lysosomes, ER, and other endomembranes. Cryofixed transformed mouse cells 6–12 μm thick were the study object. Electron microscopy images of the same structures were used as the controls. Müller et al. pointed out that membrane structures are clearly distinguishable and recognizable, since they are visually perceived in the same way as those recorded using EM, but protein components such as chromatin subdomains and nuclear bodies are much less distinguishable (which may be due to the choice of the wavelength for the image registration), not to mention ribosomes and cytoskeletal elements whose dimensions are near the limit of the instrument’s resolution. What progress has been made in the application of SXM in cell biology since this work has appeared and what results have been obtained?

A significant body of the published papers describes the application of the method to various types of cells: human and animal cells, normal or tumor cells, those infected with a virus or in contact with nanoparticles, etc. [22, 26, 27, 28, 29, 30, 31], yeast [16, 26, 32], and bacteria [12] to test the suitability of the method or a specific SXM tool for solving problems related to the visualization of subcellular structures and compare SXM with light and electron microscopy. These studies, together with “proof of concept,” propose various improvements to or combinations of methods, such as correlating a fluorescent label with an SXM image [20, 28] or combining cryo-SXT with labeling of plasma membrane proteins with colloidal gold-labeled antibodies [29].

In parallel with the development of the methodology for correlation fluorescence and SXM analysis, Duke et al. [20] also studied the system of endosomes (vesicles involved in the intracellular digestion of substances absorbed from the outside) and autophagosomes encapsulating component cells subject to recycling. To identify autophagosomes, two fluorescent genetically encoded tags were applied and the entire endocytic compartment was labeled *in vivo *with colloidal gold-labeled antibodies captured by the cell by endocytosis. In the SXM image, endosomes are identified by gold particles inside and have transparent contents. Vesicles that fall into the zones of colocalization of fluorescent labels are distinguished by dense contents and a transparent halo, and they are correlated with autophagosomes. Using the correlation of fluorescent and SXM images, specialized zones of generation of numerous autophagosome precursors (omegasomes) in the ER were visualized for the first time. Simultaneously, it was confirmed that thin and long tubular connections between individual mitochondria appear in the mitochondrial system during starvation, which are used to initiate autophagy. This example of the analysis of systemic changes in a cell not only demonstrates the power of cryo-SXT in combination with light microscopy, but also generates new data and directly corroborates the results previously obtained using other methods.

The ability of cryo-SXT to allow one to visualize the entire cell volume, identify many subcellular systems, and measure organelle sizes without using contrast/labeling was employed to study the dynamics of the redistribution of mast cell secretory granules upon antigen stimulation [33] and the emptying of insulin vesicles in secretory pancreatic cells in response to glucose stimulation [34]. It was also used to visualize and quantify pre-apoptotic changes under the influence of the anticancer agent cisplatin, in combination with adjuvants to reduce its effective concentration [35], as well as to estimate the average volume of mitochondrial fragments in cancer cells after exposure to the free radicals generated by an iridium-based photosensitizer [36]. Finally, that capacity was used to measure the mitochondrial volume, the radius of lipid droplets and cytoplasmic vesicles when cells are infected with the SaRS-CoV-2 virus [37], to analyze the redistribution of cytoplasmic vesicles and changes in the mitochondrial morphology under the influence of the herpes simplex virus [22], and to collect quantitative parameters on the response of endothelial cells to glucose stimulation (the *in vitro *model of vascular damage processes in diabetes) [38].

Along with the feasibility of carrying out morphometry at the whole-cell level, cryo-SXT allows one to capture new and often unexpected structural aspects of the studied phenomena, such as the formation of thin (at the threshold of the resolving power of CTMR), thread-like outgrowths of the ER cisterns that are formed in the contact areas between the ER and mitochondria [39]. Such areas are marked by clusters of the proteins involved in mitochondrial fission and are detected using fluorescent chimeric constructs.

Cryo-SXT was applied to visualize mitochondrial fragmentation, increase the number of lipid droplets, and cytoplasmic vacuolation in mammalian and yeast cells upon exposure to gold nanoparticles and gold ions [40, 41]. It has also been shown that a small fraction of gold nanoparticles taken up by the cell ends up in the cytoplasm rather than in the endosomallysosomal compartment. Their number is too small to be detected by their fluorescent signal and to be effectively detected in ultrathin sections by EM methods. In addition, some particles were detected in lipid droplets, which is unusual [40].

Biogenic gold nanoparticles, which are formed by yeast cells and are subsequently released into the periplasmic space, were also discovered and identified using cryo-SXT. The identification of these particles required the use of additional physical methods of measurement, and the localization of particles, including in mitochondria (for the first time), was additionally confirmed by EM [41].

The existence of such structures and phenomena cannot be assumed *a priori*; they are not resolved by light microscopy, and their accidental detection by electron microscopy is extremely unlikely or can be ignored even if it had happened.

Sometimes changes in cell physiology (e.g., the development of a pathology at the cellular level) lead to the formation of new structures with dimensions that are comparable to the cell *per se*, thus significantly impeding their detailed analysis and topology identification by EM methods, while their fine organization lies beyond the resolution of light microscopy. An example of this is the transformation of the ER when cells are infected with the hepatitis C virus, when the endoplasmic reticulum is transformed into a spongy labyrinth of membrane channels that occupy almost the entire cytoplasm [42, 43]. A detailed analysis of the geometry of membrane channels during this transformation on a cell-wide scale was carried out by cryo-SXT [30]. Changes in the contacts of the ER with mitochondria, the cell’s energy sources and important participants in lipid metabolism, which also change during viral infection, were studied simultaneously. Cryo-SXT allowed Pérez-Berná [30] to analyze the dynamics of ER transformation and show that the transformation begins locally but involves both the ER and mitochondria, starting from the onset [30].

Jamme et al. [44] used cryo-SXT to show that wild-type yeast cells and mutants producing only triacylglycerols form homogeneous lipid droplets that efficiently absorb soft X-ray photons, and, in cells producing sterol ethers only, lipid droplets have a transparent core that is surrounded by a highly absorbing shell. By combining the cryo-SXT data with the findings obtained using other non-invasive techniques, Jamme et al. confirmed the idea that lipid droplets have a layered structure with a triacylglycerol-based core and a shell formed by sterol ethers. This twolayer model was proposed based on studies of isolated lipid droplets, but the isolation procedure may have caused lipid redistribution, which did not make the result entirely convincing.

Not only organic matter density in organelles, but other factors, as well, can be an indicator of organelle composition. By varying the wavelength of soft X-ray photons, one can identify elements that have absorption peaks in the range of water transparency, estimate their concentration, and determine their state in crystalline particles and the zones of their specific concentration. This makes it possible to use SXM to study processes such as tissue mineralization, the formation of shells and spicules in invertebrates, and the absorption and excretion of nanoparticles and other nanoconstructs from cells by using the features of their composition as an additional criterion for their identification.

Thus, nitrogen distribution and the carbon-to-nitrogen ratio in the cells of *Anabena *sp. were visualized using radiation with energies greater and lower than the nitrogen absorption edge (410 eV) [45]. Under nitrogen starvation conditions, these blue-green algae form specialized cells (heterocysts) that fix atmospheric nitrogen. Using the resolving power of SXM, Teramoto et al. [45] were able to identify vegetative cells and heterocysts and analyzed the studied parameter with respect to the cell type, which had not been done previously. In both cell types, elements are distributed unevenly, the carbon-to-nitrogen ratio increases from the cell periphery towards its center, but the peripheral zone of heterocysts had a nitrogen-rich layer, which is not observed in vegetative cells.

Research into the calcium accumulation pathways (absorption edges at 352.6 and 338.3 eV) in the cells of unicellular algae forming calcite inclusions and in the mesenchymal cells of sea urchin larvae forming spicules composed of calcium carbonate showed that, despite the difference in models, both algae and echinoderm larvae have specialized vesicles that concentrate calcium ions from seawater and act as an intermediate depot for this ion [46, 47]. In algae, it is a single, large “vacuole-like” cistern [46], and the cells of sea urchin larvae contain a population of vesicles ~ 100 nm in diameter, containing calcium ion at concentrations ranging from 1 M (lower limit of detection) to a concentration corresponding to anhydrous amorphous calcium carbonate, which the spicules are composed of. SXM made it possible to accurately count the number of calcium-containing vesicles [47].

When mammalian cells absorb hydroxyapatite nanoparticles, which stimulate bone tissue regeneration, fluorescent calcium sensors show that a population of calcium-containing vesicles appears in the cells, but electron microscopy does not allow them to be identified against the background of the general population of vesicles in cells [48]. The use of cryo-SXT and analysis of the linear absorption coefficients of organelles made it possible to isolate internalized nanoparticles and identify lipid droplets and a separate population of vesicles with intermediate absorption efficiency. This correlated with multivesicular bodies; their absorption coefficient was linked to the possibility of depositing calcium ions released during nanoparticle dissolution [48].

High density and, therefore, increased contrast in cryo-SXT images is a distinctive feature of some intracellular pathogens and symbionts. This fact is efficiently used by researchers investigating the life cycles of these organisms and their interactions with cells. When studying the formation of cowpox (Vaccinia) virions and the structures that appear in infected cells, it became possible to distinguish between mature and immature virion forms and discover “viral factories” in which replication of viral genomes occurs [49].

Kördel et al. [50] studied the transfer of a substance from the host cell to the virus during the development of an unidentified giant DNA virus (presumably Cedratvirus) infecting the amoeba* Acanthamoeba castellanii*. Updated data were obtained on the number of virions formed during viral replication. Based on measurements of the absorption coefficients, the earlier estimates of material transfer obtained by less accurate and indirect methods have been corrected. It has been shown that 6–12% of the host cell substance is converted into virions. A structure has been discovered which may potentially be a viral replication factory. It has been shown that the changes affect the contractile vacuole and phagosomes, but not the nucleus, which allows the cell to function until lysis, increasing the efficiency of virus production [50]. The linear dependence of absorption coefficients on the organic matter concentration, visualized in cryo-SXT images, combined with the ability to process the entire cell volume, makes cryo-SXT a preferred tool for conducting this type of research, compared to EM, which allows the detection of much smaller virions but imposes restrictions on sample volumes. Nevertheless, EM is a necessary additional tool for this kind of work, which is directly noted by the authors of the cited publications.

Semi-automatic image segmentation, followed by measurement of the volumes of bacterial cells, showed that each intracellular “inclusion” (a vacuole in which the human pathogenic bacteria *Chlamydia trachomatis* proliferate) contains a much wider range of cell forms than estimated previously [51], according to the EM analysis of serial sections (inclusion diameter being 10–15 µm). Counting the amount of chlamydia in the inclusions showed that the volume of individual bacterial cells, which is the main criterion used to separate infectious from proliferating forms of chlamydia, depends on their concentration rather than the absolute amount. The more densely a vacuole is populated, the less often large and abnormally large cells are found in it, which means that cell concentration can be a signal of a transition from a large reproducing form to a small infectious one, all combined with host cell lysis and the next cycle of infection.

Hale et al. [52], by using inhibition assay and stopping the release of the mature asexual cells (merozoites) of *Plasmodium falciparum *from erythrocytes at various stages of this process, employed cryo-SXT as an auxiliary technique complementing both light and electron microscopy data. It was shown that the release of merozoites into the bloodstream with erythrocyte destruction, causing a fever attack in malaria, is strictly coordinated in time. Before the erythrocyte destruction, (1) disintegration of the vacuole membrane in which the merozoites are produced and release of mature merozoites into the erythrocyte cytoplasm occur, followed by (2) a collapse of the erythrocyte cytoskeleton, leading to the loss of their characteristic shape and formation of close contacts between the plasma membranes of the erythrocyte and merozoites. and (3) only then does a new generation of merozoites enter the blood. Here, cryo-SXT is a kind of control procedure supporting the light microscopy data with structural data characterized by a better resolution and allowing one to minimize interpretation errors associated with the features of sample preparation for EM.

A study of the changes at the level of individual organelles occurring in cells during the *Shigella flexneri* infection using a combination of fluorescence microscopy and cryo-SXT showed that mitochondrial fragmentation takes place in cells during the infection [53]. Correlation of light microscopy and cryo-SXT data made it possible to visualize a “trap” of septins (proteins involved in the remodeling of membranes, the cytoskeleton, and encapsulation of intracellular pathogens [54]) around Shigella cells, as well as its close connection with the autophagosome.

The study focusing on the coordination mechanisms of host and symbiont division in *Braarudosphaera bigelowii*, a unicellular alga whose cells obligately contain an endosymbiont, a cyanobacterium with a highly reduced genome that cannot exist independently but has a mechanism for nitrogen fixation, looks extremely interesting. This symbiosis may be an intermediate evolutionary phase of the symbiogenetic formation of a new organelle (a “nitroplast”), as once happened with mitochondria. The use of cryo-SXT as the main tool in this study seems quite justified. taking into account the sizes of the organism and symbiont (approximately 10 × 5 μm and 4 × 2 μm, respectively) and the high contrast between the symbiont and the chloroplasts and mitochondria of the host cell [55].

It is noteworthy that in most of the studies discussed above, the researchers focused on an inherent limitation of lipid-rich organelles: lipid droplets, mitochondria, ER cisterns, etc. Meanwhile, very little attention has been paid to such an important area as research into the ultrastructure and functioning of the cell nucleus and the genetic apparatus. This was because the efficiency of absorption of SX photons by lipids is high, making them stand out against the background of the cytoplasm [9, 11], and variations in the absorption coefficient inside the cell nucleus are insignificant; so, it becomes possible to distinguish only such large and dense formations as the nucleolus and heterochromatic blocks against the background of euchromatin [28, 56]. Meanwhile, the resolving power of the method is still insufficient in order to visualize chromatin substructures with dimensions around 100 nm or less [57]. The attempts at spectral separation of DNA, RNA, and proteins in SXM images of nuclei and chromosomes [25] offer hope for significant improvement in the contrast of nucleic acids, which will open up new opportunities in studying nuclear structures using the SXM methods. The late stages of compaction of individual chromosomes during the preparation of a eukaryotic cell for division, chromatid segregation before their distribution to daughter cells, and the initial stages of chromosome de-condensation during the formation of daughter nuclei are currently considered the most suitable for research using cryo-SXT.

**SXM and super resolution fluorescence microscopy**

Correlative light and electron microscopy, including cryo-format, are already a well-tested combination of methods [58, 59, 60]. Since cryo-SXT borrows many aspects of sample preparation from cryo-electron microscopy, it is understandable that the integration of conventional fluorescent methods and cryo-SXT has been implemented and is used to solve routine problems, primarily for localizing an object of interest with a fluorescence microscope integrated into a SXM-station before implementing cryo-SXT [11, 61]. Meanwhile, the current level of development of fluorescence microscopy in its super-resolution versions makes it possible to achieve a resolution of 100–150 nm for structured illumination microscopy and ~30 nm for SMLM and STED [1, 62], which is already comparable to the resolution of SXM.

It looks very promising to combine super-resolution light microscopy in the STED and SMLM variants with SXM. This combination of methods will potentially allow one not only to localize the molecular sources of the fluorescent signal with an accuracy of 20 nm, but also to correlate them with intracellular structures that do not carry a fluorescent label using SXM.

SMLM, combined with cryo-SXT, has been resorted to for localizing and studying the fine structure and dynamics of cholesterol crystals in a cellular model of atherosclerosis [63, 64]. Lipids have a high linear absorption coefficient in SXM [9]. So, lipid structures offer the most contrast in SXM images, but it is impossible to identify cholesterol in an overall lipid context. SMLM makes it possible to fluorescently label cholesterol and perform high-resolution studies of its distribution, albeit without reference to specific subcellular structures. With this approach, the resolution of the light component is superior to that achieved using X-ray methods: Varsano et al. [63] claimed it to be 35 nm vs. 70 nm [63].

A combination of methods made it possible to correlate the fluorescent label with structures on the plasmalemma sharply outlined in the SXM image, as well as with the surface of lipid droplets in the cytoplasm [63], and to track the movement of the crystalline structures formed by cholesterol in the cell, identifying them against the background of other lipid structures [64]. However, this integration of methods is not flawless. SMLM requires many, sometimes tens of thousands, albeit short exposures, so image registration takes considerable time. Furthermore, artificial conditions are required for implementing the mechanisms of reversible quenching and return of fluorophore molecules to the “light” state [62]. Therefore, Schermelleh [62] performed SMLM on aldehyde-fixed cells using a standard microscope, and only after that was the specimen subjected to vitrification and cryo- SXT performed. The limitations in this case include not only the need to fix the cell to implement SMLM registration, but also the fact that the carriers of fluorescence are antibodies that penetrate membranes slowly and ineffectively, which forced the authors to focus on the plasmalemma, where the target was accessible to antibodies, and make do with a low signal intensity in the cytoplasm. The combination of SMLM and cryo-SXT is attractive due to its comparable resolving power in both modalities, but the combination of limitations may be critical for further development of this approach.

The second option is to use a limited super-resolution SIM system, in combination with cryo-SXT, both image registration procedures being implemented in the cryo format. SIM allows one to work with fluorescent proteins, and, therefore, it makes it possible to directly combine light and SXM images.

The cryo format makes the use of high-aperture immersion objectives extremely difficult, although not impossible [65, 66]. To solve this problem, a specialized cryo-SIM microscope was integrated into the SXM station, in which the 3D-SIM technology using a “dry” lens with a numerical aperture of 0.9 and a large working distance allow one to obtain images with a resolution of 210 nm (diffraction limit being ~ 340 nm) without transferring heat to a sample kept at cryogenic temperatures [67].

The combination of cryo-3D-SIM and cryo-SXT was used to study the dynamics of endosomes containing reovirus particles during infection [66]. Cryo- 3D-SIM allowed the authors to visualize vesicles of various sizes and distinguish between vesicles carrying virus particles and vesicles from which the viral complex was released. The efficient rejection of outof- focus luminescence and the better axial resolution of 3D-SIM compared to diffraction-limited microscopy [62] made it possible to accurately correlate the fluorescent signal from labeled vesicles with the structures observed in the cryo-SXT image. The key observation was that the egress of the virus does not disrupt the endosomes, leaving their shape round and their membranes intact. As suggested [62], virions can leave endosomes due to the formation of collapsible pores in the membranes.

3D-SIM was used to localize bundles of actin filaments in the SXM image. These filaments poorly absorb X-ray photons and are, therefore, virtually invisible; however, highly detailed fluorescent data allows one to accurately identify the zone where the actin structures are located and identify their intracellular environment using SXM [68].

To solve similar problems related to correlating fluorescence and X-ray images, a unique “laser scanning confocal cryo-tomograph” with an immersion lens was built using a full-rotating cryo-sample holder from a complementary cryo-SXT instrument [28]. The microscope was tested on a model of localization and visualization of the Barr body (inactivated X chromosome) in female mouse cells and became an essential part of the SXM station at the ALS synchrotron (USA) [39].

An obvious application of this combination of methods is the identification of X-ray images of intracellular structures that have either been poorly characterized or not characterized at all, for which protein markers are available, especially in connection with work focusing on creating “X-ray atlases” of cell morphology [9, 11]. Despite the general similarity of EM and SXM images, not all structures are displayed in the same way due to differences in sample preparation. All that is left to do is wait for the design of a cryo-3D-SIM installation with an immersion lens integrated into the SXM station.

## PROSPECTS FOR SXM

**A promising SXM tool**

An alternative to the optical design with zone plates, which has become the industry standard in cryo- SXT, can be the use of normal-incidence mirror lenses optimized for the wavelengths of the water transparency window [69]. Calculations show that such a microscope, with a completely achievable numerical aperture of 0.3 [70] (vs. ~0.05–0.06 for the zone plates) at a wavelength of 3.37 nm will allow for a lateral resolution of ~ 5 nm, which is an order of magnitude better than the standard achievable 40–70 nm in cryo-SXT and lies in the range previously accessible only by EM. This tool is currently a laboratory prototype and has not yet reached its design parameters; however, the functionality of all elements of the system has been demonstrated.

An inherent limitation of this instrument is the anisotropic resolution associated with stretching of the point spread function along the main optical axis of the device, like in a visible light microscope [71]. The same simple calculation shows that the axial resolution of such an instrument will approach 40 nm, which is somewhat better than when working on an EM in normal mode, where the axial resolution is equal to the thickness of the physical slice, very rarely reaching 50 nm (usually thicker) (*Fig. 1*). The shallow depth of focus of this SXM significantly complicates the implementation of angular tomography, so that for three-dimensional reconstruction, simpler algorithms for volume reconstruction from a series of optical sections (z-tomography) using deconvolution [72, 73, 74], like for widefield and confocal visible light microscopes, become optimal. Proper choice of the deconvolution algorithm and its parameters can partially compensate for the resolution anisotropy, but eliminating it completely will be impossible because of its fundamental nature.

Based on this optical scheme, a design for an SXM station was proposed [71] for the SKIF synchrotron source (“Siberian Circular Photon Source”) that is currently under construction. The project involves two operating modes: scanning using a lens with a numerical aperture of 0.3 to illuminate the sample with a beam focused onto a spot of diffraction dimensions, with registration of variations in the brightness of the passing beam with a single-pixel detector when scanning by displacing the sample relative to the beam, and a widefield mode in which a second lens builds an image sample on the matrix detector. It is planned that the instrument will be equipped with a cryostage for biological applications.

Potentially, due to its short focal distance and the small thickness of the optical section and high lightcollecting ability, which in turn reduces the radiation dose required to obtain an image, this SXM in the widefield mode will allow one to observe dynamic processes in living cells isolated from vacuum in an enclosed fluidic microchamber. The question of the practical feasibility of such observations remains open.

**Selective labels for SXM**

Light microscopy offers a wide array of methods for highly selective labeling of subcellular structures and biomolecules (fluorescently labeled antibodies, constructs with fluorescent proteins, high-specificity fluorescent dyes, etc.) (*Fig. 1A,D*). In contrast, electron microscopy largely relies on morphological criteria, accumulated through the decades of EM development, for identifying the observed structures. Selective labeling techniques in EM are less varied and not as reliable as those in light microscopy. SXM in general and cryo-SXT, during its development, may turn out to be in the same situation.

To be compatible with cryofixation, selective imaging techniques should not affect cell viability when functioning *in vivo*. If any auxiliary action is required to visualize the labeled structure (e.g., fixation), the question immediately arises: is the “native state” of the cell preserved? Moreover, such a label must efficiently generate contrast when using radiation in which the biological matter is transparent and, therefore, must differ drastically from the “living matter” in terms of structure or composition. In the study by Kong et al. [75], a protein localization system developed for EM based on chimeric constructs with peroxidases and photoactivatable proteins that generate reactive oxygen species was adapted for SXM. Visualization of the label is achieved by oxidation of diaminobenzidine (DAB), facilitated by the labeled protein. DAB penetrates the plasmalemma of both fixed and living cells [76]. Its insoluble oxidized form is locally deposited and efficiently absorbs soft X-ray photons due to the high density of the DAB precipitate [75]. The technique involves aldehyde fixation before “developing” the label with a solution of DAB and hydrogen peroxide; however, unlike the EM version of this method, the SXM version does not require additional contrasting with OsO4 [76]. The use of the free radical generator protein miniSOG, instead of peroxidase, does not require incubation with hydrogen peroxide; it is activated by irradiation with visible light [75]. Obviously, the protocol can be combined with other proposed labeling systems [20, 63].

## CONCLUSION

With all its features and the relatively “young age” of the method, soft X-ray microscopy (primarily in the implementation of cryo-tomography) has productively been added to the toolkit of cell biology. The most promising direction for its further development seems to be closer integration of cryo-SXT imaging and super resolution light microscopy. The development of genetically encoded tags, especially “multimodal” ones, capable of generating both a fluorescent signal and absorption contrast in SX, will add wholly new possibilities to this promising combination of methods, allowing researchers to switch from correlation and purely morphological criteria in ultrastructural analysis to direct consideration of the molecular aspects of the structure and dynamics of subcellular systems. We remain hopeful that the current technical challenges discussed above will be overcome and such a combined approach will become practically accessible.

It can be envisioned that in the future there will be a closer convergence of SXM with cryo-EM. For example, integration of a focused ion beam source into a cryo-SXT instrument will enable cryo-SXT navigation to produce lamellae for cryo-EM tomography including objects of interest. Ideally, this integration will result in a “seamless pipeline” that allows comprehensive interrogation of cell structure and function across a full range of scales and resolutions, leveraging the strengths of all the available microscopic techniques.

The main “infrastructural” problems of cryo-SXT that remain unresolved are the needfor expensive equipment for sample cryofixation during its preparation and a limited number of SXM installations at large synchrotron sources, each of which is unique in its own way. These factors limit the affordabilty of the method. The construction of new X-ray sources, including SKIF installations with an already planned X-ray microscopy station and RIF (“Russian Photon Source”) in the Russian Federation, will lead to a significantly deeper integration of SXM into research practice.

The emergence of sufficiently bright laser-plasma sources of soft X-ray photons has spurred attempts to create “laboratory-level” devices [31, 32, 77] to uncouple SXM instruments from the “mega-science” installations. So far, these efforts are of a design and exploratory nature, but the creation of a commercially distributed SXM, comparable in price and operating costs to biological EM, should make SXM methods as popular as light and electron microscopy. Designing a laboratory cryo-SXT microscope with high-aperture optics and a resolution of 5–7 nm can potentially dislodge EM from the position of the primary method of ultrastructural imaging, leaving it only as a segment of near-atomic resolution.

## Abbreviations

cryo-SXT: cryotomography in the soft X-ray range
SXM: soft X-ray microscopy
EM: electron microscopy (microscope)
SMLM: single-molecule localization microscopy
STED: stimulated emission depletion
SIM/3D-SIM: structured illumination microscopy
ER: endoplasmic reticulum
